# Piwi-interacting RNAs play a role in vitamin C-mediated effects on endothelial aging

**DOI:** 10.7150/ijms.42586

**Published:** 2020-03-26

**Authors:** Sulin Zheng, Haoxiao Zheng, Anqing Huang, Linlin Mai, Xiaohui Huang, Yunzhao Hu, Yuli Huang

**Affiliations:** 1 Department of cardiology, Shunde hospital, Southern Medical University (The first people's hospital of Shunde, Foshan), Guangdong, China; 2 Second Medical College of Southern Medical University, Guangzhou, China

**Keywords:** aging, endothelial cells, vitamin C, Piwi-interacting RNAs

## Abstract

The underlying mechanisms that mediate the effects of vitamin C on endothelial cell aging are widely unknown. To investigate whether Piwi-interacting RNAs (piRNAs) are involved in this process, an endothelial aging model was induced* in vitro* using H_2_O_2_ in human umbilical vein endothelial cells (HUVECs) and then treated with vitamin C (VC). Untreated HUVECs without H_2_O_2_ exposure were used to serve as the negative control group. Cell cycle, cell viability, and aging-associated protein expression were assessed, and RNA sequencing was performed to reveal the piRNA profile. Functional and regulatory networks of the different piRNA target genes were predicted by the Kyoto Encyclopedia of Genes and Genomes (KEGG) pathway enrichment and Gene Ontology (GO) analysis. H_2_O_2_ induced G1 phase cell arrest, decreased cell viability, and upregulated the senescence marker p16 in HUVECs. We found that VC treatment inhibited G1 phase cell arrest, increased the number of cells in the S and G2/M phases, increased cell viability, and decreased p16 expression. The piRNA expression profiles revealed that a large proportion of piRNAs that were differentially expressed in H_2_O_2_-treated HUVECs were partly normalized by VC. Furthermore, a number of piRNAs associated with the response to VC in H_2_O_2_-treated HUVECs were linked with senescence and cell cycle-related pathways and networks. These results indicate that the ability of VC to attenuate H_2_O_2_-mediated endothelial cell senescence may be associated with changes in expression of piRNAs that are linked to the cell cycle.

## Introduction

The morbidity and mortality rate of cardiovascular disease (CVD) is relatively high worldwild 1. Cellular senescence is an irreversible arrest of cell growth. Accumulating evidence suggests that cellular senescence exerts important effects on vascular endothelial dysfunction, which is believed to be an early pathological event during CVD development. For example, secretion of atherosclerosis-related factors, such as intercellular adhesion molecule-1 and the inhibitory activity of endothelial nitric oxide synthase, is found in senescent human umbilical vein endothelial cells (HUVECs) 2. Atrial endothelial senescence was found to promote thrombus formation and extracellular matrix remodeling 3. In an aging mouse model with vascular endothelium damage, antioxidant therapy can alleviated the dysfunction of aging vascular endothelium cells 4. Studies have shown that long-term addition of the antioxidant vitamin C (VC), which can reduce the immune levels associated with thymic aging 5. VC may also be involved in the function of cardiovascular endothelial cells 6, which can improve endothelial dysfunction in patients with hypertension 7. However, the mechanisms of VC in cardiovascular aging-related endothelial injury are still unclear.

Piwi-interacting RNAs (piRNA) are a type of non-coding RNA of approximately 26-32 nucleotides in length 8. They maintain genomic integrity by regulating the expression of retrotransposons 9, 10. piRNA is widely expressed in adult cells, including germ cells, cardiomyocytes, and cerebral vascular endothelial cells, and it is involved in regulating CVD-related pathways 11-14. However, the expression pattern of piRNA in the context of cardiovascular endothelial cell senescence remains unknown.

Herein, we investigated the effect of VC on H_2_O_2_-induced senescence in endothelial cells and whether piRNA is involved in VC-mediated antiaging effects.

## Materials and Methods

### Cell culture and drug treatment

HUVEC (PUMC-HUVEC-T1) were purchased from the Cell Resource Center of IBMS, CAMS/PUMC (Beijing, China). Cells were cultured in Dulbecco's Modified Eagle's Medium (DMEM; Invitrogen, Waltham, MA, USA) containing 10% fetal bovine serum and incubated in a 5% CO_2_ incubator at 37°C. Cells were treated with H_2_O_2_ (100 μM) to create an *in vitro* model of senescence (H_2_O_2_ group) 15-17. After 2 h, some of these cells were then cultured in medium containing VC (200 μM; Sigma-Aldrich, St. Louis, MO, USA) to investigate the effect of VC on aging endothelial cells (VC group). Cells without H_2_O_2_ or VC treatment were used as negative controls (Control group).

### Flow cytometry

Approximately 10^6^ cells for each treatment condition were collected and suspended in cold PBS. Cells were then incubated in 70% ethanol overnight. The following day, cells were incubated in propidium iodide (BD Biosciences, San Jose, CA, USA) and subjected to flow cytometry (Becton, Dickinson and Company, Franklin Lakes, NJ, USA) to determine the stages of cell cycle. DNA content was calculated at each phase of the cell cycle.

### [3-(4,5-dimethylthiazol-2-yl)-5-(3-carboxymethoxyphenyl)-2-(4-sulfophenyl)-2H-tetrazolium] (MTS) assay

Cells were grown in a 96-well-plate at a density of 1×10^4^/well and incubated in 10% DMEM containing 20 μL of Cell Titer 96 AQueous One Solution Reagent (Promega, Madison, WI, USA). Following incubation for 1 h, cell viability was assessed by determining absorbance at 450 nm using a 96-well plate reader (Bio-Rad Laboratories, Hercules, CA, USA).

### Western blot analysis

To explore the effect of VC on HUVECs senescence, the expression of p16, a marker of cellular senescence 18-20 was detected by Western blot analysis. After H_2_O_2_ and VC treatment, cells were harvested and lysed using RIPA buffer (Beyotime Biotechnology, Shanghai, China). Proteins were quantified using a Bradford assay kit (Pierce, Rockford, IL, USA) and were resolved on a 10% SDS-polyacrylamide gradient gel by electrophoresis. Proteins were then transferred to polyvinylidene fluoride membranes (Millipore, Burlington, MA, USA). The membranes were incubated in 5% bovine serum albumin for 1 h. To detect p16 expression, anti-p16 antibody (10883-1-AP, Proteintech, Rosemont, IL, USA, diluted 1:1000) was added to the membranes and incubated at 4°C overnight. Then, the membranes were incubated with secondary HRP-conjugated antibody (ab6721, Abcam, Cambridge, MA, USA, diluted 1:2000) at room temperature for 1 h. Horseradish peroxidase (HRP) signal was detected using a chemiluminescence reagent (Millipore, Burlington, MA, USA). Protein expression was quantified using ImageJ software (version 1.8.0, National Institutes of Health, Bethesda, MD, USA). GADPH (Abcam, Cambridge, MA, USA) was used as the internal control.

### RNA sequencing

TRizol reagent (Thermo Scientific, Waltham, MA, USA) was used to isolate total RNA from the cells. Small RNA libraries were generated using the TruSeq small RNA library preparation kit (Illumina, San Diego, CA, USA) according to the manufacturer's recommendation. RNA sequencing was performed on an Illumina HiSeq 2000 platform (Illumina, San Diego, CA, USA) for small RNA sequencing. Cutadapt (version 1.8.1, National Bioinformatics Infrastructure Sweden, Uppsala, Sweden) were used to remove low-quality reads (sequences containing the linker, >10% N bases, or >50% bases with a <10 mass value) from resulting raw data, the remaining sequences were further aligned against the human genome (hg19). Reads that were between 26 and 32 nucleotides in length met the criteria for piRNAs 8.

### Bioinformatics analysis

An R package (version 3.1, Lucent Technologies, Reston, VA, USA) was used to analyze the differentially expressed piRNAs. piRNAs that were differentially expressed following H_2_O_2_ treatment with or without VC were defined using a fold change of > 2 and Benjamini-Hochberg false discovery rate of < 0.001. An R package was used to predict candidate target mRNA for the piRNAs that were ranked in the top 10 for fold change and were restored by VC. Candidate mRNA and piRNA interaction networks were drawn using Cytoscape software (version 3.7.2, Cytoscape Consortium, San Diego, CA, USA).

To predict the function of the piRNAs involved in the effect of VC, Kyoto Encyclopedia of Genes and Genomes (KEGG) pathway enrichment (https://www.genome.jp/kegg/) and Gene Ontology (GO) analysis (http://geneontology.org/) were performed using an R package according to accepted standards.

### Statistical analysis

Data were expressed as mean ± standard deviation. Statistical analysis were performed using SPSS software (version 10.0, International Business Machines Corporation, Armonk, NY, USA). Comparison of differences between groups was performed using one-way analysis of variance with Fisher LSD post-hoc tests. A *p* value of < 0.05 was considered significant.

## Results

### VC alleviates H_2_O_2_-mediated endothelial cell senescence

**Figure 1:**
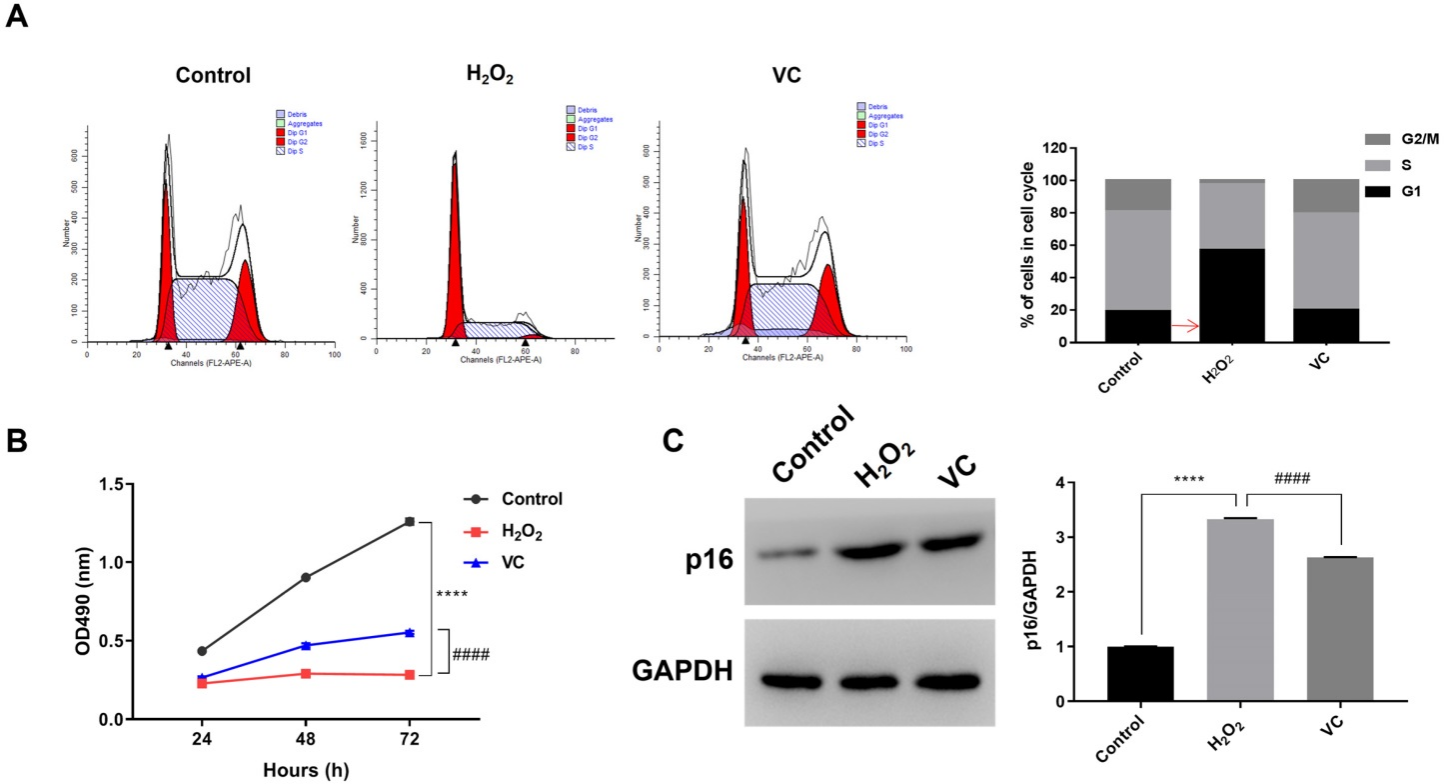
** Effects of VC on H_2_O_2_-induced senescence in endothelial cells.** (a) Flow cytometry showing the DNA content in each phase of the cell cycle, normalized to total content and expressed as a percent of cells in each cell cycle. (b) MTS assay showing the dynamic changes in cell viability 72 h after VC treatment. (c) Quantification of p16 protein expression after VC treatment. Control group, HUVEC cells without any treatment; H_2_O_2_ group, HUVEC cells exposed to H_2_O_2_; VC group, HUVEC cells exposed to H_2_O_2_ followed by treatment with VC. **** *p* < 0.0001, H_2_O_2_ group vs control group; #### *p* < 0.0001, VC group vs H_2_O_2_ group.

Flow cytometry analysis showed that, compared with the control group, H_2_O_2_ treatment increased the proportion of the cells in G1 phase, resulting in a decrease in the proportion of cells in the S phase and G2/M phase. Conversely, VC treatment in H_2_O_2_-exposed cells significantly increased the proportion of cells in the S phase and G2/M phase (Fig. 1a). MTS assay revealed that, compared with the control group, exposure to H_2_O_2_ (H_2_O_2_ group) for 72 h resulted in a significant decrease in cell viability, whereas cells treated with VC after H_2_O_2_ exposure (VC group) had significantly higher cell viability than the H_2_O_2_ group (Fig. 1b). In addition, western blot analysis showed that the expression of p16 was significantly elevated after H_2_O_2_ exposure, and treatment with VC can reduce the expression of p16 induced by H_2_O_2_ exposure (Fig. 1c).

### piRNA expression profile following VC treatment

**Figure 2:**
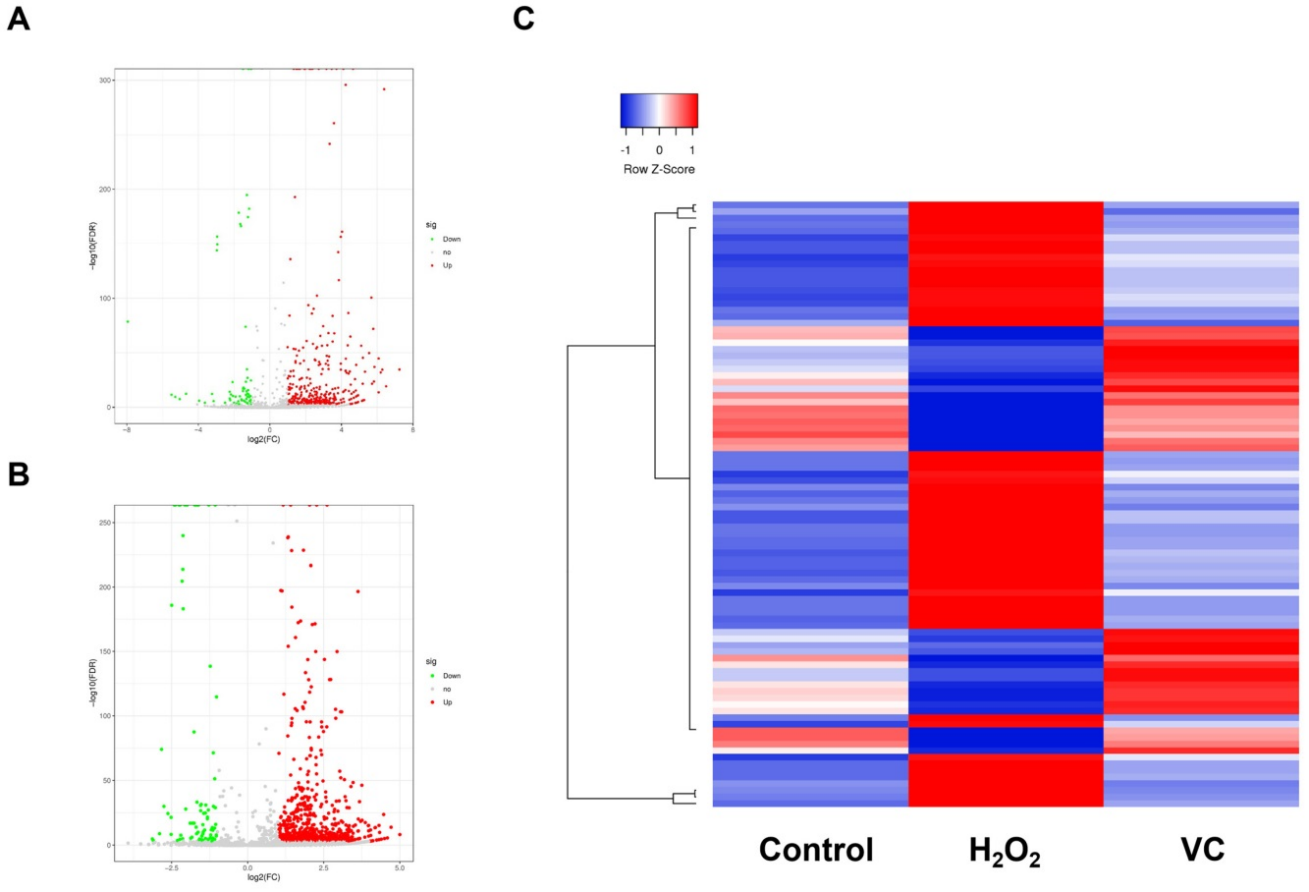
** Volcano plot (a, b) and hierarchical clustering analysis (c) revealing the differentially expressed piRNAs that were observed after H_2_O_2_ induction and recovery by VC.** Control group, HUVEC cells without any treatment; H_2_O_2_ group, HUVEC cells exposed to H_2_O_2_; VC group, HUVEC cells exposed to H_2_O_2_ followed by treatment with VC.

RNA-seq was performed to characterize the piRNA expression in control, H_2_O_2_, and VC groups respectively. As shown in Fig. 2a, a volcano plot revealed that many piRNAs were differentially expressed in the H_2_O_2_ group compared with the control group. Of these, 340 piRNAs were upregulated, and 62 piRNAs were downregulated (Fig. 2a). The VC group also had significantly different expression levels of many piRNAs (Fig. 2b). There were 36 upregulated and 56 downregulated piRNAs in the VC group compared with the H_2_O_2_ group. Unbiased hierarchical clustering analysis showed a sequence of piRNAs that were upregulated after H_2_O_2_ induction and downregulated when VC was added to the cells. Conversely, some piRNAs were downregulated after H_2_O_2_ induction and were restored after VC treatment (Fig. 2c).

### Potential role of aging-associated and VC-responsive piRNAs

**Figure 3:**
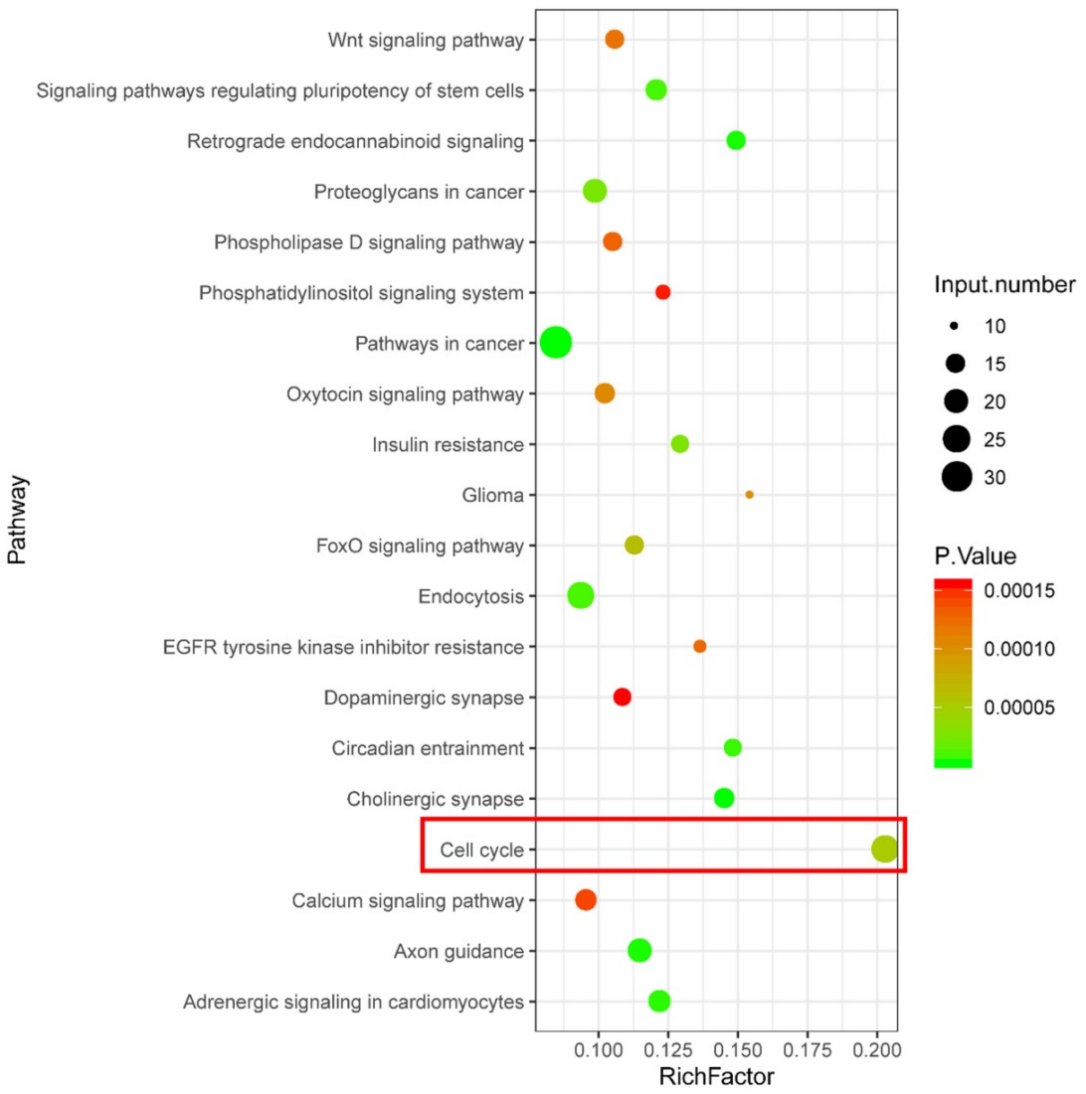
KEGG enrichment analysis showing signaling pathways related to potential function of differentially expressed piRNA target genes.

KEGG pathway enrichment analysis was performed using the target genes of differentially expressed piRNAs that were abnormally regulated in the H_2_O_2_ group and restored in the VC group. As shown in Fig. 3, KEGG analysis revealed that a series of piRNA target genes were enriched in the cell cycle pathway.

**Figure 4:**
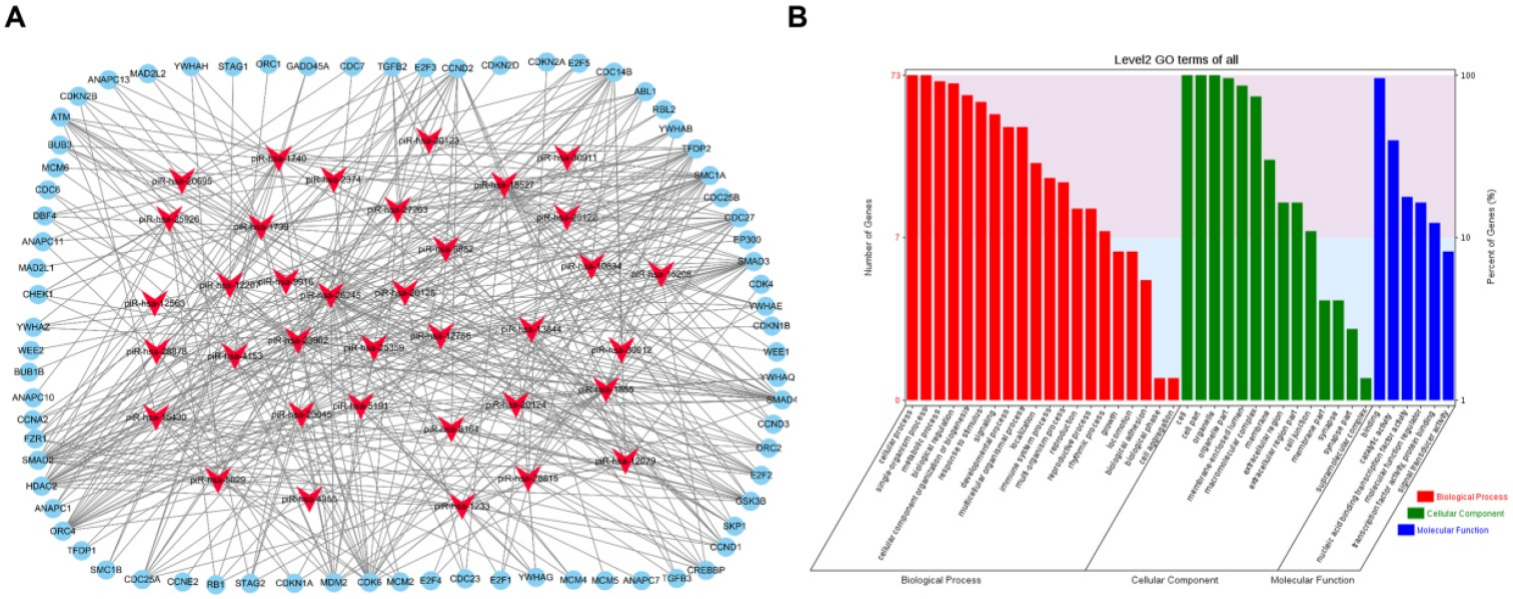
** Functional analysis of VC-associated piRNAs.** (a) piRNA-mRNA regulatory network, mRNA were enriched in cell cycle pathway in KEGG analysis, piRNAs of those upregulated by H_2_O_2_ and recovered by VC treatment (b) GO analysis predicting the potential role of piRNA target genes in (a).

**Figure 5:**
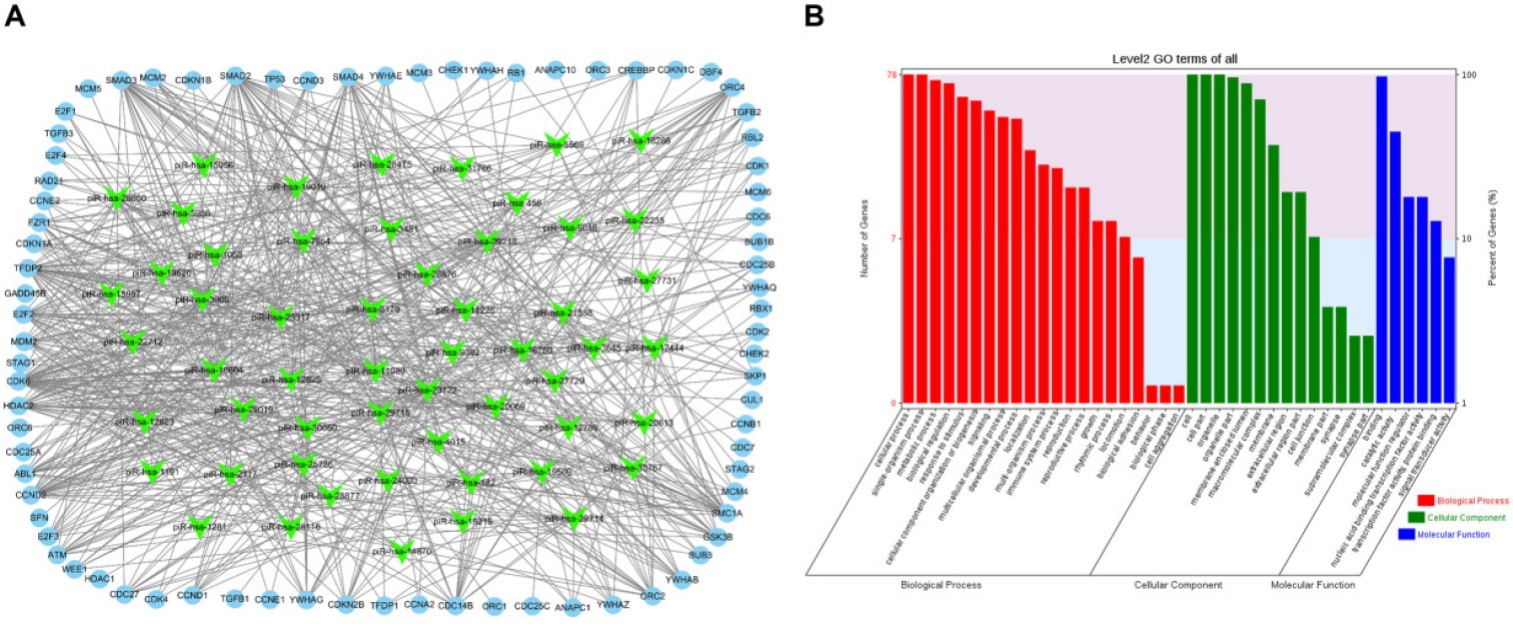
** Functional analysis of VC-associated piRNAs.** (a) piRNA-mRNA regulatory network, mRNA were enriched in cell cycle pathway in KEGG analysis, piRNAs were those downregulated by H_2_O_2_ and rescued by VC treatment (b) GO analysis predicting the potential role of piRNA target genes in (a).

To visualize the regulatory network of piRNAs restored by VC treatment in the cell cycle pathway, two piRNA-mRNA networks, one for the upregulated piRNAs and one for the downregulated piRNAs, were generated. The upregulated piRNA-mRNA network consisted of 36 piRNAs and 73 cell cycle-associated mRNAs (Fig. 4a), and the downregulated network consisted of 56 piRNAs and 78 cell cycle-associated mRNAs (Fig. 5a). GO analysis of the target genes of these piRNAs revealed that the top two terms within each block were cellular process, single-organism process, cell, cell part, binding, and catalytic activity (Fig. 4b and 5b).

## Discussion

The present study assessed the protective effects of VC in the H_2_O_2_ model of cellular senescence in HUVECs. H_2_O_2_ treatment promoted cell arrest in the G1 phase, inhibited cell proliferation, and increased expression of aging markers. VC treatment attenuated the effects of H_2_O_2_ on cell cycle, cell proliferation, and expression of cell senescence markers. Further analysis of the piRNA expression profiles in the control group, H_2_O_2_ group, and VC group demonstrated that VC can repair the altered piRNA expression profile induced by H_2_O_2_. Moreover, bioinformatics analysis showed that the protective effect of VC on the H_2_O_2_ group is related to the cell cycle.

When cells are stimulated by external factors, they may exhibit protective responses such as apoptosis, autophagy, and aging. Damaged endothelial cells are more likely to age than undamaged cells 21. One of the manifestations of the cellular aging response to stress is G1 arrest. This change in cell cycle inhibits the proliferation of damaged cells and prevents potential malignant cellular transformations. Studies have shown that the decline in yeast cell proliferation caused by aging and cell death is associated with G1/S phase transition disorders 22. Decreased cell proliferation levels and G1 phase cell arrest were also observed in senescent breast cancer cells 23. These studies suggest that the blockade of the G1/S phase transition and decreased cell proliferation levels are among the hallmarks of cellular senescence. In addition to cell cycle transformation disorders, increased expression of the cell senescence marker p16 can be observed 23. Previous studies have suggested that p16 inhibits function of cyclin-dependent kinases, causing G1 cell cycle arrest 18. The current study used the oxidant H_2_O_2_ to observe the effects of VC on cell cycle and cell viability. H_2_O_2_ induced a decrease in endothelial cell viability and inhibition of G1/S transformation, whereas VC treatment ameliorated the senescence-associated profile. These results suggest that VC has a role in alleviating the H_2_O_2_-induced cellular senescence phenotype.

The current study also found that the effect of VC on HUVECs treated with H_2_O_2_ is related to piRNAs. Prior to the present report, only one study demonstrated the presence of piRNAs in prostate epithelial tissue from a vitamin D supplementation trial, suggesting that piRNA expression may be associated with the effects of vitamins 24. The current study provides evidence of piRNA expression related to VC. Furthermore, the functional prediction of piRNA target genes that were normalized following VC treatment revealed that these targets are enriched in numerous signaling pathways including cell cycle, endocytosis, circadian entrainment, Wnt signaling pathway, Forkhead box O (FoxO) signaling pathway, cholinergic synapse, oxytocin signaling pathway, axon guidance, and adrenergic signaling in cardiomyocytes. Among these pathways, the FoxO signaling pathway notably mediates oxidative stress and prolongs lifespan 25, 26. The oxytocin signaling pathway may play a role in regulating CVD and age-related disorders 27, 28. In particular, cell cycle is a significant pathway that corresponds with the observed improvement of G1/S transition in VC-treated cells. Therefore, we speculate that the function of some piRNAs may associate with the effect of VC in H_2_O_2_-treated cells, while more experimental evidence is needed in future studies.

By constructing the piRNA-mRNA interaction networks, the current study demonstrated that VC-mediated regulation of endothelial function-associated piRNAs regulates a range of cell cycle-related genes. However, the precise function of these differentially expressed piRNAs is still unknown. Among the targets of the piRNAs within the network, *CDK2* is an important gene that regulates the cell cycle and promotes cell cycle transition by binding to different ligands. For example, *CDK2* binding to cyclin E can phosphorylate retinoblastoma protein to promote the G1/S phase transition 29, 30. It also has been found that *MDM2* promotes the G1/S transition that is suppressed by p53 31. Furthermore, *MYC* functions to promote cell cycle progression, and inhibition of *MYC* expression can lead to G1 arrest 32, 33. In addition, *HDAC1* is necessary to suppress G1/S phase transitions 34. Finally, *MCM* is a gene that rests during G1, and it is activated after cells enter the S phase. *MCM* plays an important role in the replication of S phase cells 35. Therefore, we propose that the piRNAs dysregulated by H_2_O_2_ were protected by VC treatment, and their association with the cell cycle may facilitate G1/S transition that was suppressed by H_2_O_2_ and may contribute to delays in cellular senescence.

In summary, the current study found that VC plays a role in repairing the H_2_O_2_-induced aging phenotype of vascular endothelial cells and may have a protective effect on aging-related CVD. Furthermore, this VC-induced antiaging phenotype may be related to piRNA expression. These piRNAs were primarily enriched in antiaging, metabolic, and aging-related cardiovascular regulatory pathways. This study supports the hypothesis that piRNAs play a significant role in the effect of VC against endothelial aging.

## References

[1] Townsend N, Wilson L, Bhatnagar P, Wickramasinghe K, Rayner M, Nichols M (2016). Cardiovascular disease in Europe: epidemiological update 2016. European heart journal.

[2] Minamino T, Miyauchi H, Yoshida T, Ishida Y, Yoshida H, Komuro I (2002). Endothelial cell senescence in human atherosclerosis: role of telomere in endothelial dysfunction. Circulation.

[3] Hasan H, Abbas M, Auger C, Belcastro E, Farooq MA, Park SH (2018). Atrial endothelial cells senescence promotes thrombogenicity, inflammation and extracellular matrix remodeling: Role of the local Ang II/AT1 receptor pathway. Archives of Cardiovascular Diseases Supplements.

[4] Bhayadia R, Schmidt BMW, Melk A, Hömme M (2015). Senescence-induced oxidative stress causes endothelial dysfunction. Journals of Gerontology Series A: Biomedical Sciences and Medical Sciences.

[5] Uchio R, Hirose Y, Murosaki S, Yamamoto Y, Ishigami A (2015). High dietary intake of vitamin C suppresses age-related thymic atrophy and contributes to the maintenance of immune cells in vitamin C-deficient senescence marker protein-30 knockout mice. British Journal of Nutrition.

[6] Qin X, Qin L, Luo J, Liu B, Zhao J, Li H (2019). Correlation analysis between 25-hydroxyvitamin D3, vitamin B12 and vitamin C and endothelial function of patients with CHD. Experimental and therapeutic medicine.

[7] Solzbach U, Hornig B, Jeserich M, Just Hr (1997). Vitamin C improves endothelial dysfunction of epicardial coronary arteries in hypertensive patients. Circulation.

[8] Vella S, Gallo A, Nigro AL, Galvagno D, Raffa GM, Pilato M (2016). PIWI-interacting RNA (piRNA) signatures in human cardiac progenitor cells. The international journal of biochemistry & cell biology.

[9] Aravin A, Gaidatzis D, Pfeffer S, Lagos-Quintana M, Landgraf P, Iovino N (2006). A novel class of small RNAs bind to MILI protein in mouse testes. Nature.

[10] Moyano M, Stefani G (2015). piRNA involvement in genome stability and human cancer. Journal of hematology & oncology.

[11] Girard A, Sachidanandam R, Hannon GJ, Carmell MA (2006). A germline-specific class of small RNAs binds mammalian Piwi proteins. Nature.

[12] Sharma AK, Nelson MC, Brandt JE, Wessman M, Mahmud N, Weller KP (2001). Human CD34+ stem cells express the hiwigene, a human homologue of the Drosophila genepiwi. Blood.

[13] Yin K-J, Hamblin M, Chen YE (2014). Non-coding RNAs in cerebral endothelial pathophysiology: emerging roles in stroke. Neurochemistry international.

[14] Rajan KS, Velmurugan G, Pandi G, Ramasamy S (2014). miRNA and piRNA mediated Akt pathway in heart: antisense expands to survive. The international journal of biochemistry & cell biology.

[15] Hernandez-Segura A, de Jong TV, Melov S, Guryev V, Campisi J, Demaria M (2017). Unmasking transcriptional heterogeneity in senescent cells. Current Biology.

[16] Ruan Y, Wu S, Zhang L, Chen G, Lai W (2014). Retarding the senescence of human vascular endothelial cells induced by hydrogen peroxide: effects of 17beta-estradiol (E2) mediated mitochondria protection. Biogerontology.

[17] Du L, Chen E, Wu T, Ruan Y, Wu S (2019). Resveratrol attenuates hydrogen peroxide-induced aging through upregulation of autophagy in human umbilical vein endothelial cells. Drug design, development and therapy.

[18] Rayess H, Wang MB, Srivatsan ES (2012). Cellular senescence and tumor suppressor gene p16. International journal of cancer.

[19] Krishnamurthy J, Torrice C, Ramsey MR, Kovalev GI, Al-Regaiey K, Su L (2004). Ink4a/Arf expression is a biomarker of aging. The Journal of clinical investigation.

[20] He S, Sharpless NE (2017). Senescence in Health and Disease. Cell.

[21] Vicencio JM, Galluzzi L, Tajeddine N, Ortiz C, Criollo A, Tasdemir E (2008). Senescence, apoptosis or autophagy?. Gerontology.

[22] Neurohr GE, Terry RL, Sandikci A, Zou K, Li H, Amon A (2018). Deregulation of the G1/S-phase transition is the proximal cause of mortality in old yeast mother cells. Genes & development.

[23] Alipoor FJ, Asadi MH, Torkzadeh-Mahani M (2018). MIAT lncRNA is overexpressed in breast cancer and its inhibition triggers senescence and G1 arrest in MCF7 cell line. Journal of cellular biochemistry.

[24] Baumann B, Lugli G, Gao S, Zenner M, Nonn L (2019). High levels of PIWI-interacting RNAs are present in the small RNA landscape of prostate epithelium from vitamin D clinical trial specimens. The Prostate.

[25] Lehtinen MK, Yuan Z, Boag PR, Yang Y, Villén J, Becker EBE (2006). A conserved MST-FOXO signaling pathway mediates oxidative-stress responses and extends life span. Cell.

[26] Mouchiroud L, Houtkooper RH, Moullan N, Katsyuba E, Ryu D, Cantó C (2013). The NAD+/sirtuin pathway modulates longevity through activation of mitochondrial UPR and FOXO signaling. Cell.

[27] Wang P, Wang SC, Yang H, Lv C, Jia S, Liu X (2019). Therapeutic Potential of Oxytocin in Atherosclerotic Cardiovascular Disease: Mechanisms and Signaling Pathways. Frontiers in neuroscience.

[28] Cho SY, Kim AY, Kim J, Choi DH, Son ED, Shin DW (2019). Oxytocin Alleviates Cellular Senescence through Oxytocin Receptor-Mediated ERK/Nrf2 Signalling. British Journal of Dermatology.

[29] Pines J (1991). Cyclins: wheels within wheels. Cell Growth & Differ.

[30] Wingren AG, Nyesiga B (2018). CDK2 (cyclin dependent kinase 2). Atlas of Genetics and Cytogenetics in Oncology and Haematology.

[31] Chen J, Wu X, Lin J, Levine AJ (1996). mdm-2 inhibits the G1 arrest and apoptosis functions of the p53 tumor suppressor protein. Molecular and cellular biology.

[32] Bretones G, Delgado MD, León J (2015). Myc and cell cycle control. Biochimica et Biophysica Acta (BBA)-Gene Regulatory Mechanisms.

[33] Jeong YJ, Hoe HS, Cho HJ, Park KK, Kim DD, Kim CH (2018). Suppression of c-Myc enhances p21WAF1/CIP1-mediated G1 cell cycle arrest through the modulation of ERK phosphorylation by ascochlorin. Journal of cellular biochemistry.

[34] Micheli L, D'Andrea G, Leonardi L, Tirone F (2017). HDAC1, HDAC4, and HDAC9 bind to PC3/Tis21/Btg2 and are required for its inhibition of cell cycle progression and Cyclin D1 expression. Journal of cellular physiology.

[35] Wei L, Zhao X (2016). A new MCM modification cycle regulates DNA replication initiation. Nature structural & molecular biology.

